# Tunable heat shock protein-mediated NK cell responses are orchestrated by STAT1 in Antigen Presenting Cells

**DOI:** 10.1038/s41598-021-95578-3

**Published:** 2021-08-09

**Authors:** Abigail L. Sedlacek, Lauren B. Kinner-Bibeau, Yifei Wang, Alicia P. Mizes, Robert J. Binder

**Affiliations:** 1 Department of Immunology, University of Pittsburgh School of Medicine, University of Pittsburgh, E1058 Biomedical Science Tower, 200 Lothrop Street, Pittsburgh, PA 15261 USA; 2 KromatiD Inc, Longmont, CO 80501 USA; 3 Tsinghua University, Medical School,, Beijing, 100084 China; 4 Department of Medicine, University of Pittsburgh, Pittsburgh, PA 15261 USA

**Keywords:** Chemokines, Cytokines, Signal transduction, Dendritic cells, Monocytes and macrophages

## Abstract

The release of Heat Shock Proteins (HSPs) from aberrant cells can initiate immune responses following engagement of the HSPs with antigen presenting cells (APCs). This is an important mechanism for cancer immunosurveillance and can also be modeled by vaccination with HSPs through various routes, targeting specific APCs expressing the HSP receptor CD91. Immunological outcomes can be varied as a result of the broad expression of CD91 in different dendritic cell and macrophage populations. We investigated the cellular response of different APCs to the prototypical immunogenic HSP, gp96, in the context of Th1 immunity. Although APCs generally express similar levels of the HSP receptor CD91, we uncovered APC-distinct, downstream signaling pathways activating STAT1, and differential STAT1 induced genes. As a result of this differential and unique signaling we determined that gp96-activated macrophages, but not DCs are capable of activating NK cells to produce IFN-$\gamma$. These data demonstrate that different APC subsets elicit unique intracellular signaling responses to HSPs which result in different patterns of downstream cellular activation and immune responses. Collectively this provides a novel tunable and autochthonous immune response to extracellular HSPs which has important implications on the development of immunity to cancer and infectious disease, as well as homeostasis.

## Introduction

Antigen presenting cells (APCs) are sentinel immune cells in tissues with a primary function of recognizing aberrant cells arising as a result of infection, malignancy or trauma. An important and evolutionarily conserved pathway for recognizing such aberrancy is through the detection of extracellular HSPs, which are released by cells through a variety of mechanisms including necrosis^1^. We have shown that detection of certain extracellular HSPs, including gp96, hsp90, hsp70 and calreticulin, by APCs occurs via the cell surface receptor LRP1/CD91^2,3^. Mechanistically, CD91 allows for the endocytosis of HSPs and the cross-presentation of HSP-chaperoned peptides^4–6^. In addition, CD91 initiates signaling cascades that culminate in the release of cytokines and upregulation of co-stimulatory molecules^1^. These events lead to priming of innate and adaptive immune responses that are important in rejection of cancers and maintenance of tissue homeostasis^7^. The basis for these immunological outcomes derives from the CD91^+^ APC that engages the extracellular HSPs^8^.

APCs, including dendritic cells and macrophages and their respective subsets, located in a variety of tissues, express CD91 and have the capacity to bind and respond to extracellular HSPs^2^. We have shown that the type of APC that engages HSPs dictates the type and magnitude of the immune response^8,9^. When gp96 engages classical type 1 DCs (cDC1s), immune responses generated are of the Th1 type, which are capable of rejecting tumors^4,9,10^. However, gp96 can also engage plasmacytoid DCs (pDCs) to elicit regulatory T cell (Treg) responses that can prevent or revert Th1 mediated (auto)immunity^8,11–13^. These and other responses to extracellular HSPs are a result of APC-specific signaling and epigenetic modifications within the APC. CD91 has two NPXY motifs which are phosphorylated in response to HSP binding. Phosphorylation of the membrane proximal Tyr is necessary for activation of NF-$\upkappa$B and p38 MAPK^14^. Although additional signaling proteins and transcription factors have not yet been identified, we have shown that, at minimum, such signaling through CD91 is necessary for the release of cytokines^14^. Downstream, these cytokines subsequently have consequences for T cell priming and, as shown recently, for activation of NK cells^15^. Soluble factors such as CXCL10, CCL5, IL-18, and IL-15 are known activators of NK cells^16–18^, released by APCs in response to HSPs^15^ and, controlled at the transcriptional level, in mice and humans, by STAT1^19^. We thus investigated STAT1 phosphorylation in a variety of APCs in response to HSPs, to understand the regulation of molecules that are important for NK cell activation.

In this study, we show that while both macrophages and cDCs express CD91 to similar levels, macrophages rapidly phosphorylate STAT1 in response to gp96 whereas DCs experience a protracted delay. This leads to a reduced expression of STAT1-induced genes by DCs including the NK cell activating chemokine, CXCL10. The functional consequences of this differential STAT1 signaling in APCs, in response to gp96, yield macrophages but not cDCs are capable of activating NK cells as measured by IFN-$\gamma$ production. We show that the difference between macrophages and cDCs to phosphorylate STAT1 is, at least in part, due to increased levels and activity of the phosphatase SHP2 in DCs. Inhibition of SHP2 partially restores rapid STAT1 phosphorylation in cDCs to levels similar to macrophages. This regulation of signaling networks in different APCs provides a tunable and specific immune response towards a particular insult. Additionally, these signaling cascades act with similarity to and/or in synergy with others such as the IFN system, where type I and type II interferons activate the same pathways. However, differences in kinetics and magnitudes result in different immunological consequences^20,21^. Since the release of HSPs can occur in any tissue following cellular aberrancy in response to infection, malignancy or trauma, understanding how diverse tissue-specific APCs respond to extracellular HSPs is important for therapeutic manipulation of immune responses for clinical benefit.

## Materials and methods

All methods below were carried out in accordance with the relevant guidelines and regulations as outlined by the University of Pittsburgh.

### Animals

Female C57BL/6 mice, 6–8 weeks old, were purchased from The Jackson Laboratory (Bar Harbor, ME). The mice were housed in our DLAR facilities and used under experimental protocols approved by the University of Pittsburgh Institutional Animal Care and Use Committee (IACUC). STAT1 KO mice were a kind gift from Dr. John Alcorn (University of Pittsburgh). All animal experiments were carried out in compliance with ARRIVE guidelines.

### Cell culture

All cells were cultured in RPMI containing 1% sodium pyruvate, 1% L-glutamine, 1% nonessential amino acids, 1% penicillin and streptomycin, 0.1% 2-mercaptoethanol, and 10% heat inactivated FBS (GIBCO). Adherent PECs (referred to as PECs) were generated as previously described^15^. Briefly, the peritoneal cavity of naïve mice was lavaged with 4 mL sterile PBS. Cells were plated so that the final cell densities would be as indicated in each experiment, taking into consideration that 35% of the harvested cells are adherent (Supplemental Fig. 5). Total isolated cells were plated overnight at 37 °C and non-adherent cells were removed the following day. BMDCs were generated by culturing 5 × 10^6^ bone marrow cells in 20 ng/mL GMCSF (R&D) for 6–7 days, supplementing RPMI and GMCSF on day 3. Non-adherent cells (BMDCs) were plated at the indicated densities for individual assays. SpDCs and NK cells were isolated from the spleens of naïve mice using the MACS pan DC isolation kit and NK cell isolation kit II (Miltenyi Biotech), respectively.

Unless otherwise indicated, 5 × 10^4^ APCs were cultured with 5 × 10^4^ NK cells in 96-well flat bottom plates for 24 or 72 h in the presence of 200 $\upmu$g/mL gp96 or or 1 $\upmu$g LPS. The following neutralizing antibodies/inhibitors were used at the indicated concentrations in some experiments; $\mathrm{\alpha }$-CXCR3(CXCR3-173)/hamster IgG: 100 $\upmu$g/mL (ebioscience), $\mathrm{\alpha }$-IL-18(93-10C)/rat IgG: 10 $\upmu$g/mL (MBL), $\mathrm{\alpha }$-IL-18R(AF856)/goat IgG: 10 $\upmu$g/mL (R&D), $\mathrm{\alpha }$-CCL5(MAB478)/rat IgG: 7 $\upmu$g/mL (R&D), DAPTA: 10 nM (Abcam), CXCL10: 0.5–50 ng/mL (R&D). Neutralizing antibodies/inhibitors were added at the same time as gp96 and maintained throughout the co-culture duration. Culture supernatants were harvested and used as samples in the Femto-HS IFN-$\gamma$ ELISA (eBioscience/Invitrogen). SB203580 (EMD Millipore) was used at a final concentration of 10 $\upmu$M and was added to APCs 2 h prior to gp96 activation and maintained throughout activation. SHP099 (Cayman Chemical) was used at a final concentration of 100 mM and was added to APCs 3 h prior to gp96 activation and maintained throughout activation.

### Protein analysis

Gp96, calreticulin, and HSP90 were isolated from murine livers as previously described^10,22^. The quality and purity of the gp96 preparations was vigilantly verified by SDS-PAGE gel and immunoblotting and is consistent with our previous studies.

For immunoblots, cells were lysed with NP-40 and lysates were run on SDS-PAGE gels and transferred to nitrocellulose membranes. Membranes were probed with the following antibodies $\mathrm{\alpha }$-STAT1(14944), $\mathrm{\alpha }$-pSTAT1 S727(9177), $\mathrm{\alpha }$-pSTAT1 Y701(9167), $\mathrm{\alpha }$-pp38 MAPK(9167), $\mathrm{\alpha }$-p38 MAPK(9216), $\mathrm{\alpha }$-$\upbeta$-actin(8457), (Cell Signaling) and $\mathrm{\alpha }$-SHP2(sc-7384) (Santa Cruz). Immunoblots comparing different cell types and timepoints were run with all on a singular membrane when possible. Membranes were developed using a Proteinsimple imager and quantitated using Alpha View software.

Luminex (eBioscience) consisted of the single analytes: IL-12, IL-18, IL-15/IL-15R, IFN-$\mathrm{\alpha }$, IP-10, and RANTES. Samples were analyzed using the manufacturer’s protocol and a Bioplex II Luminex machine.

CXCL10 and CCL5 ELISAs were purchased from eBioscience and samples were analyzed following the manufacturer’s protocol.

### Flow cytometry

Cells were fixed with BD-Cytofix/Cytoperm for 20 min at 4 °C, but not resuspended in permeabilization buffer. Following fixation, cells were stained with $\mathrm{\alpha }$-CD91 (clone 5A6) in PBS with 1% BSA and 0.1% sodium azide. An $\mathrm{\alpha }$-mouse IgG-FITC antibody was used for secondary staining. Samples were run on a BD LSR II and analyzed using Flow Jo (Tree Star).

### STAT1 chromatin immunoprecipitation

Adherent PECs were stimulated for 1 h with 200 µg/ml gp96 or equivalent volume PBS in at a density of 1 × 10^6^ cells in a 24-well plate. Cell lysis and chromatin immunoprecipitation was done using the ChIP-IT Express Kit (Active Motif) following the manufacturer’s suggested protocol. Lysates were sonicated using Bioruptor pico (Diagenode) and were sonicated to 100–300 base pair fragments as determined by gel electrophoresis. Chromatin was immunoprecipitated using anti-STAT1(14,994) (Cell Signaling) or isotype antibodies. Anti-STAT1, isotype, and input samples were analyzed by qPCR using the following primers designed against the CXCL10 promoter: (Forward primer: CCATGGTTAGAACCTGACTTAG; Reverse primer: GCAGTGCCTTGCAGAATA). Quantification was performed by normalizing STAT1 precipitated samples to input. Percent input was calculated by: % input = 2deltaCTx100.

### Statistical analysis

Statistical analysis was run using Graph Pad Prism software version 7. Student’s t-test or ANOVA were used where indicated. P < 0.05 was considered significant.

## Results

### Macrophages and DCs have differential capacities to activate NK cells

**Figure 1:**
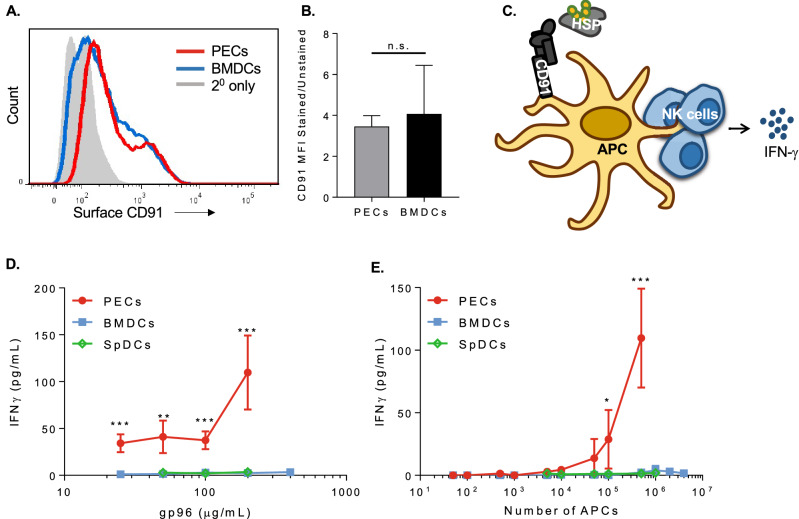
PECs but not BMDCs are capable of inducing NK cell IFN-$\gamma$ production in response to gp96. **(A** and **B)** PECs and BMDCs were stained for CD91 and analyzed by flow cytometry. **(A)** A representative histogram is shown and **(B)** MFI is pooled from 3 independent experiments and graphed. **(C)** Illustrated setup of in vitro co-culture assay. **(D** and **E)** Equivalent numbers of PECs, BMDCs, or SpDCs were co-cultured with 5 × 10^4^ NK cells and the indicated dose of gp96 for 24 h. Supernatants were assayed for IFN-$\gamma$ secretion by ELISA. **(D)** Numbers of antigen presenting cells were titrated using a constant 200 $\upmu$g/mL concentration of gp96. **(E)** Concentration of gp96 was titrated using a constant (5 × 10^5^) number of APCs. Data are represented as mean ± s.d, *p < 0.05, **p < 0.01, ***p < 0.001 (Student’s t-test, B or One-way ANOVA, D and E). In D and E, data are pooled from at least 3 independent experiments is shown.

Primary adherent peritoneal exudate cells (PECs) and bone marrow derived dendritic cells (BMDCs) were used as representative macrophage and dendritic cells respectively. Both cell types express CD91 to similar levels (Fig. 1A,B) and are known to respond to gp96 by releasing IL-1$\upbeta$ and IL-6^1,14^. Using a co-culture system previously characterized by us^15^ (Fig. 1C), we tested the ability of PECs and BMDCs to activate NK cells in response to titrated doses of gp96 (Fig. 1D) and across a wide spectrum of APC densities, up to 4 × 10^﻿6^ cells (Fig. 1E). IFN-$\gamma$ was measured as an indication of NK cell activation. While PECs activated NK cells across all gp96 doses tested and with as few as 10^5^ PECs, BMDCs were not observed to activate NK cells at any dose of gp96 tested and even with 4 × 10^6^ BMDCs. In addition to gp96, we tested two additional immunogenic HSPs, calreticulin and HSP90, to activate PEC and BMDC co-cultures. Similar to activation with gp96, PECs but not BMDCs induced NK cell IFN-$\gamma$ production in response to the other HSPs. As a positive control, the TLR2/4 ligand LPS stimulated PECs and BMDCs to induce NK cell activation (Supplemental Fig. 1). Given the unexpected results obtained in BMDCs, we verified our results with primary DCs purified from spleens (spDCs). Similar to BMDCs, spDCs also failed to activate NK cells in response to HSPs.

In the co-culture systems used in Fig. 1, there is no IFN-$\gamma$ release in the absence of APCs (gp96 + NK cells only) consistent with the observation that NK cells do not express CD91 or respond directly to gp96. Also, in the absence of NK cells (gp96 + APC only), no IFN-$\gamma$ is detected. Furthermore, flow cytometric analysis of the co-cultured cells shows only the only IFN-γ + cells are also NK1.1 + ^15^.

### Gp96 orchestrates release of cytokines and chemokines that are unique to the APC

**Figure 2:**
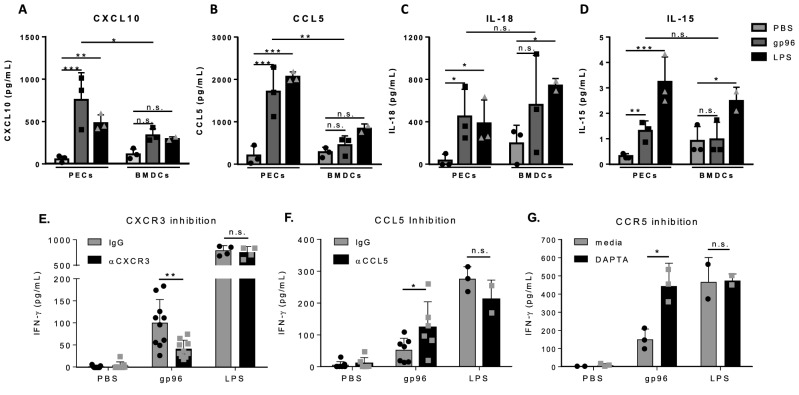
CXCR3 ligands are required for NK cell activation by PECs. (**A–D)** One million PECs or BMDCs were incubated with 200 $\upmu$g/mL gp96 or 1 $\upmu$g LPS for 10 h. Supernatants were harvested and analyzed by Luminex for the secretion of **(A)** CXCL10 **(B)** CCL5 **(C)** IL-18 and **(D)** IL-15. **(E–I)** Fifty thousand PECs were plated with 5 × 10^4^ NK cells in the presence of 200 $\upmu$g/mL gp96 and **(E)** $\mathrm{\alpha }$-CXCR3 antibody, **(F)** $\mathrm{\alpha }$-CCL5 antibody, or **(G)** DAPTA. Supernatant was harvested after 72 h and assayed by IFN-$\gamma$ ELISA. Data in all panels are pooled from 3 independent experiments. Data are represented as mean ± s.d. *ns* not significant, *p < 0.05, **p < 0.01, ***p < 0.001 [Two-way ANOVA with Sidak’s multiple comparison test (all panels) and Dunnett’s multiple comparison test (A-D)].

To understand why PECs were superior to BMDCs at activating NK cells, we tested the capacity of PECs and BMDCs to release CXCL10, CCL5, IL-12, IL-15, IL-18 and IFN-$\mathrm{\alpha }$; cytokines and chemokines known to activate NK cells^16–18^. Equal numbers of PECs and BMDCs (1 × 10^6^) were activated with 200 $\upmu$g/mL gp96 for 10 h. Supernatants were harvested and analyzed by luminex for the indicated cytokines and chemokines (Fig. 2A-D). PECs released significantly more CXCL10 (Fig. 2A) and CCL5 (Fig. 2B) than BMDCs. No significant differences in IL-18 (Fig. 2C) or IL-15 (Fig. 2D) were detected in cultures of PECs versus BMDCs activated with gp96. IFN-$\mathrm{\alpha }$ and IL-12p70 were undetectable in either cell type except in response to the positive control LPS (Data not shown; limit of detection = 4 pg/mL and 2.5 pg/ml respectively). Importantly, several responses are distinct between gp96 and LPS, for example, IL-15 (Fig. 2D PECs = p < 0.01, BMDCs = p < 0.05 not shown), IFN-$\mathrm{\alpha }$, IL-12 (data not shown) demonstrating that gp96-mediated activation via CD91 is not identical to TLR2/4 mediated activation.

We next tested the requirement of CXCL10 and CCL5, chemokines differentially expressed by PECs and BMDCs in response to gp96, for NK cell activation by their inhibition. The cell culture system from Fig. 1C was set up with PECs (as the APC) and with the inclusion of inhibitors to chemokines (CCL5) and/or chemokine receptors (CXCR3, CCR5). Inhibition of the CXCL10 receptor, CXCR3, significantly reduced the activation of NK cells by PECs (Fig. 2E). This reduction was not due to differences in NK cell proliferation or death (Supplemental Fig. 2A–C). Inhibition of CCL5 (Fig. 2F) or its receptor, CCR5 with DAPTA (Fig. 2G), did not abrogate NK cell activation by PECs and in fact increased IFN-$\gamma$ production. Although CCL5 has other receptors besides CCR5, they are not expressed by NK cells. PECs and BMDCs released similar levels of IL-18 in response to gp96, and its inhibition did not interfere with NK cell activation (Supplemental Fig. 2D–E). LPS-mediated IFN-$\upgamma$ production by NK cells was reduced upon IL-18/IL-18R inhibition, again emphasizing the differences between TLR4(LPS) and CD91(gp96) -mediated mechanisms for NK cell activation.

### Selective phosphorylation of STAT1 by gp96

**Figure 3:**
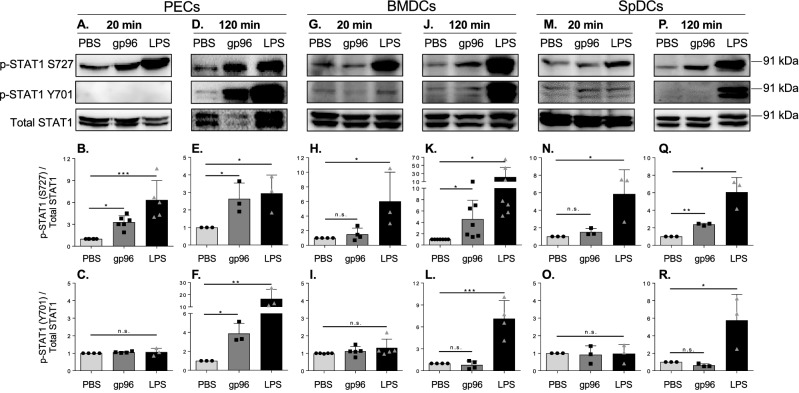
Differential STAT1 phosphorylation in PECs and DCs in response to gp96. **(A–F)** PECs, **(G–L)** BMDCs, or **(M–R)** SpDCs were activated with 200 $\upmu$g/mL gp96 for **(A–C, G–I, M–O)** 20 or **(D–F, J–L, P–R)** 120 min. Cells lysates were obtained, analyzed by SDS-PAGE and immunoblotted for total STAT1 and phospho-STAT1 at residues S727 and Y701. Immunoblot data **(A, D, G, J, M, P)** shown represents one of at least 3 independent experiments. Data in the graphs **(B–C, E–F, H–I, K–L, N–O, Q–R)** are pooled from at least 3 independent experiments. Data are represented as mean ± s.d. *ns* not significant. *p < 0.05, **p < 0.01, ***p < 0.001 (one-way ANOVA with Sidak’s multiple comparison test).

CXCL10 is a STAT1 induced gene^23^. We examined the phosphorylation of STAT1 in PECs and BMDCs in response to gp96. STAT1 has two phosphorylation sites; ser727 (S727) and tyr 701 (Y701). S727, but not Y701, was phosphorylated in PECs after 20 min of stimulation (Fig. 3A-C). Both sites were phosphorylated at the 2 h time point (Fig. 3D-F). In BMDCs, neither the S727 nor Y701 site were phosphorylated at 20 min (Fig. 3G-I). S727 was phosphorylated after 2 h (Fig. 3J,K) but no phosphorylation of Y701 was observed for up to 2 h (Fig. 3L). Phosphorylation of STAT1 in spDCs mirrored STAT1 phosphorylation in BMDCs; neither S727 (Fig. 3M,N) nor Y701 (Fig. 3M,O) were phosphorylated at 20 min and only S727 (Fig. 3P,Q), not Y701 (Fig. 3P,R), was phosphorylated by 2 h. In both PECs and BMDCs, we used LPS as a stress inducer of STAT1 and were able to induce phosphorylation, although with different kinetics than gp96 (Fig. 3G,H,J,L). Both BMDCs and spDCs responded to LPS similarly to PECs suggesting that they do not have in intrinsic defect in phosphorylating STAT1 in response to all STAT1 stress-inducers, but instead in the selective response to gp96.

**Figure 4:**
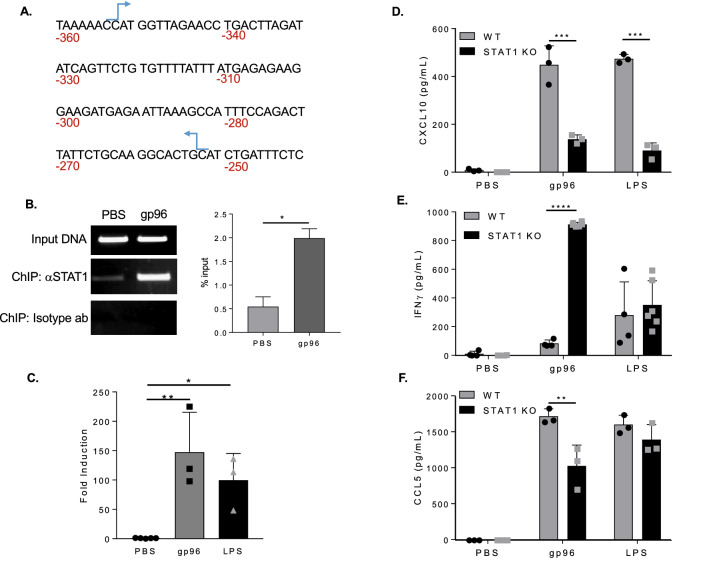
STAT1 is required for CXCL10 production by PECs. **(A and B)** PECs were treated with 200 $\upmu$g/mL gp96 for 1 h. STAT1-bound DNA was isolated by ChIP from cell lysates. Isolated DNA was amplified by RT qPCR for CXCL10 **(A)** Illustration of the promoter region of CXCL10. Primers sites used for amplification are indicated by the arrows. **(B)** Percent input was calculated for 3 independent experiments and also analyzed on an EtBr gel. **(C)** PECs were treated with 200 $\upmu$g/mL gp96 for 8 h. Cells were lysed and RNA was harvested for qRTPCR. Fold change in expression of CXCL10 mRNA was determined relative to the PBS treated sample in each cell type. **(D)** WT or STAT1 KO PECs were activated with 200 g/mL gp96 for 24 h. Supernatants were assayed by ELISA for the secretion of CXCL10. **(E)** WT or STAT1 KO PECs were co-cultured with WT NK cells in the presence of 200 μg/mL gp96. IFN-$\gamma$ secretion was measured by ELISA. **(F)** WT or STAT1 KO PECs were treated as (D) and CCL5 release was measured by ELISA. Data in the graphs data are pooled from 3 independent experiments. Data are represented as mean ± s.d. *ns* not significant, *p < 0.05, **p < 0.01, ***p < 0.001 (student’s t-test used in panel B; one-way ANOVA with Sidak’s multiple comparison test used in panel C-F).

Phosphorylation of STAT1 leads to homo- and heterodimerization, nuclear translocation, DNA binding and upregulation of targeted genes^24^. Phosphorylation of S727 and Y701 are required to mediate these effects. To determine if gp96-mediated phosphorylation of STAT1 was sufficient to result in STAT1 binding to a target promoter, here CXCL10, we performed a ChIP assay. PECs were pulsed with gp96 for 1 h. This time point is sufficient for S727 phosphorylation and is the earliest we can observe phosphorylation of Y701 (Fig. 3A-F, and Supplemental Fig. 3). Chromatin was isolated, immunoprecipitated with STAT1-specific antibodies, and subjected to PCR with primers spanning the indicated region in the promoter of CXCL10 (Fig. 4A). Results show STAT1 was bound to the promoter of CXCL10 (Fig. 4B), suggesting that in response to gp96, phosphorylation of STAT1 is sufficient for translocation into the nucleus and binding to the indicated promoter region. We monitored the corresponding expression of the CXCL10 gene following STAT1 binding. RNA was harvested from cell lysates and assayed by qRT-PCR for the expression of CXCL10 mRNA. In Fig. 4C, we show an increase in CXCL10 mRNA, in accord with the increased protein expression observed in Fig. 2. Additionally, we investigated the necessity of STAT1 on CXCL10 production by PECs. WT and STAT1 KO PECs were activated with gp96 for 24 h and supernatants were assayed for CXCL10 release. STAT1 KO PECs released significantly less CXCL10 in response to gp96 when compared to WT cells (Fig. 4D). To see if this reduction in CXCL10 production translated to decreased IFN-$\gamma$ production by NK cells, we co-cultured STAT1 KO PECs and WT NK cells in the presence of gp96. Unexpectedly, STAT1 KO PECs induced significantly more IFN-$\gamma$ production than WT PECs (Fig. 4E). Gp96-activated PECs produce both activators (i.e. CXCL10) and inhibitors (i.e. CCL5) of NK cell IFN-g production and the balance of these determine the extent of NK cell IFN-$\gamma$ production (Fig. 2E-G). We investigated the level of CCL5 released by gp96 activated STAT1 KO PECs. Similar to the levels of CXCL10, CCL5 release was significantly decreased in STAT1 KO PECs (Fig. 4F), suggesting that lack of STAT1 leads to an extensive dysregulation of multiple factors that influence NK cell activation.

### Gp96-mediated signaling activates STAT1 via p38

**Figure 5:**
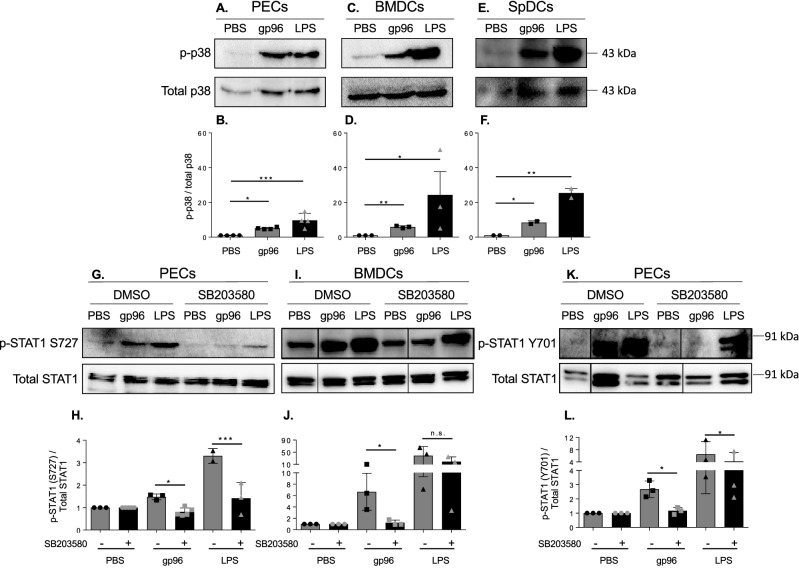
P38 expression does not differ among APC and is required for STAT1 phosphorylation. **(A-F)** PECs, BMDCs, or SpDCs were activated with 200 $\upmu$g/mL gp96 or 1 $\upmu$g LPS for 20 min. Cells lysates were immunoblotted for phospho-p38 as well as total p38. **(A,C,E)** Representative blots are shown. **(B,D,F)** Pooled data from 3 independent experiments is shown. **(G-K)** PECs or BMDCs were pre-treated for 2 h with SB203580 and then activated with 200 $\upmu$g/mL gp96 or 1 $\upmu$g LPS for **(G-H)** 20 min or **(I-L)** 120 min. Cells lysates were immunoblotted for phospho-STAT1 **(G-J)** S727 or **(K-L)** Y701 as well as total STAT1. Immunoblot data **(A,C,E,G,I,K)** shown represents one of at least 3 independent experiments. Data in the graphs **(B,D,F,HJ,L)** are pooled from at least 3 independent experiments. Data are represented as mean ± s.d. *ns* not significant, *p < 0.05, **p < 0.01, ***p < 0.001 (One-way ANOVA used in panels B,D,F; and Two-way ANOVA used in panels H,J,L; both with Sidak’s multiple comparison test).

STAT1 stress-inducers such as LPS, UV irradiation, and TNF-$\mathrm{\alpha }$, generally signal STAT1 S727 phosphorylation through p38^25^. We investigated the role of p38 in STAT1 phosphorylation in the response of PECs and BMDCs to gp96. PECs and BMDCs were activated with gp96 for 20 min, upon which they were harvested and probed for p-p38 and total p38. Similar levels of p38 phosphorylation were observed in PECs (Fig. 5A,B) and BMDCs (Fig. 5C,D). We confirmed this observation in spDCs. Similar levels of p38 phosphorylation were detected in spDCs following stimulation with gp96 (Fig. 5E,F). LPS was used as a positive control for p38 phosphorylation in all 3 cell types. We tested the dependence of STAT1 phosphorylation on p38 by using the inhibitor SB203580. We assessed the phosphorylation of STAT1 S727 in PECs at 20 min (Fig. 5G,H) and BMDCs at 120 min (Fig. 5I,J). These time points are the earliest times at which we observe S727 phosphorylation in PECs and BMDCs respectively. STAT1 S727 phosphorylation in PECs and BMDCs following stimulation with gp96 was inhibited by SB302580 (Fig. 5H,J). The mechanism of STAT1 Y701 phosphorylation in response to stress-inducers is yet undefined and it is not known whether this is controlled by p38. To determine if phosphorylation of Y701 in response to gp96 is p38 mediated, we also measured Y701 phosphorylation in PECs in the presence of SB203580. Gp96-mediated phosphorylation of Y701 was also inhibited by SB203580 (Fig. 5K,L). Importantly LPS-mediated phosphorylation of Y701 was not dependent on p38, suggesting gp96, LPS, and other stress-inducers of STAT1 phosphorylation may induce phosphorylation through different mechanisms.

### BMDCs express increased active SHP2 which impedes STAT1 S727 phosphrylation in response to gp96

**Figure 6:**
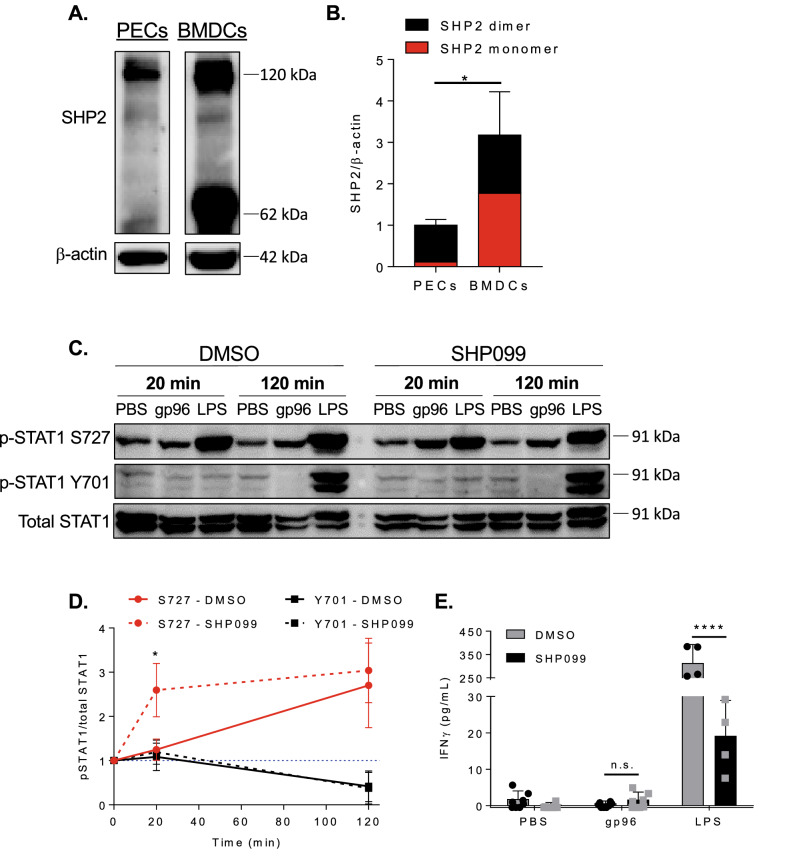
SHP2 limits STAT1 phosphorylation in DCs. **(A** and **B)** Cell lysates from PECs or BMDCs were immunoblotted for SHP2. **(A)** One representative immunoblot of 3 independent experiments is shown. Both PEC and BMDC samples were run on the same blot and developed together. **(B)** Pooled data from 3 independent experiments is shown. * indicates significance between PECs and BMDCs in the amount of monomer present. **(C** and **D)** BMDCs were pre-treated for 3 h with SHP099 then activated with 200 $\upmu$g/mL gp96 or 1 $\upmu$g LPS for 20 or 120 min. Cell lysates were immunoblotted for phospho-STAT1 S727 or Y701 and total STAT1. **(C)** One representative immunoblot of 3 independent experiments is shown. **(D)** Pooled data from 3 independent experiments is shown. * indicates significant difference in S727 phosphorylation between DMSO and SHP099 treated cells. **(E)** Fifty thousand PECs were plated with 5 × 10^4^ NK cells in the presence of 200 $\upmu$g/mL gp96 and SHP099. Supernatant was harvested after 72 h and assayed by IFN-$\gamma$ ELISA. Data is pooled from 3 independent experiments. Data are represented as mean ± s.d. *p < 0.05, **p < 0.01, ***p < 0.001 (Two-way ANOVA with Sidak’s multiple comparison test).

P38 is phosphorylated in BMDCs and is responsible for the eventual phosphorylation of STAT1 S727, thus we predicted another factor was inhibiting earlier phosphorylation of S727, specifically in BMDCs and not in PECs. SHP2 is a phosphatase known to regulate STAT1 phosphorylation at both the Tyr and Ser sites^26^. In addition, SHP2 is recruited to the phosphorylated NPXY motifs of CD91^27^ and activated in response to CD91 ligands such as PDGF^28^. Thus, we examined the steady state SHP2 protein levels present in PECs and BMDCs by immunoblotting (Fig. 6A,B). BMDCs have approximately threefold more total SHP2 than PECs. In addition, approximately 50% of the SHP2 found in BMDCs is present as a monomer, whereas the SHP2 in PECs exists almost exclusively in the dimeric form which is enzymatically inactive^29^ (Fig. 6B). Combined, this results in a net 15 fold higher amount of active SHP2 in BMDCs compared to PECs. We next tested whether inhibition of SHP2 would result in earlier phosphorylation of STAT1 S727 and eventual phosphorylation of Y701 in BMDCs, similar to the kinetics in PECs. We abrogated SHP2 function with the allosteric inhibitor SHP099^30^. Inhibition of SHP2 resulted in phosphorylation of STAT1 S727 at 20 min in BMDCs (Fig. 6C,D), similar to phosphorylation in PECs (Fig. 3B). In contrast, SHP099 had no effect on STAT1 Y701 phosphorylation. To determine if this partial rescue of STAT1 phosphorylation is sufficient to induce NK cell IFN-$\gamma$ production, we performed BMDC-NK cell co-culture experiments in the presence of SHP099. SHP2 inhibition was not sufficient to induce NK cell activation in BMDC co-cultures (Fig. 6E), suggesting that the inability for BMDCs to activate NK cells is dependent on Y701 phosphorylation. Y701 phosphorylation in PECs is thus necessary for NK cell activation and also suggests other differences between PECs and BMDCs contribute their differential phosphorylation of STAT1 and unique responses to gp96.

## Discussion

Our findings uncover a novel tunable system involving endogenous molecules released from cells that defines the immunological phenotype. Extracellular HSPs are capable of engaging CD91 expressed on APCs. Depending on the histological location of the release of HSPs, APCs in that locale may be of a wide variety including macrophage and DC subsets. We have used PECs, BMDCs and spDCs as a representation of diverse APC subsets that express CD91 and examined how they respond to one immunogenic HSP, gp96, in the context of their ability to activate NK cells. Although both PECs and BMDCs signal through CD91 and phosphorylate p38, only PECs progress to STAT1 phosphorylation, resulting in the increased expression of STAT1 regulated genes such as CXCL10. In contrast, BMDCs express higher levels of the phosphatase SHP2 which inhibits STAT1 phosphorylation. Functionally, CXCL10 produced by PECs, in response to gp96, is necessary for NK cell activation. BMDCs fail to activate NK cells in response to gp96.

One of the chief differences we determined between macrophages and DCs is the expression of the phosphatase SHP2, also known as PTP11. Although SHP2 is classically identified as a tyrosine phosphatase for a number of substrates, SHP2 is capable of regulating both serine and tyrosine phosphorylation of STAT1 in whole cell lysates^26^. In our studies, inhibiting SHP2 with SHP099 did not result in increased phosphorylation of STAT1 at Y701, but it did increase phosphorylation of STAT1 at S727 in BMDCs at early time points. We postulate that other tyrosine phosphatases are involved in regulating phosphorylation of Y701. In support of this reasoning, BMDCs treated with pervanadate, a selective Tyr phosphatase inhibitor exhibit increased STAT1 Y701 phosphorylation basally and further increases in response to gp96 (Data not shown).

The pattern and mechanism of STAT1 phosphorylation in response to gp96 is distinct from the conventional STAT1 phosphorylation described for IFN signaling. Following IFN receptor signaling through the JAK and/or TYK adaptor proteins, STAT1 is phosphorylated at Y701, dimerizes with itself or other STAT proteins, translocates to the nucleus, and binds to promoter sequences^31^. Subsequent phosphorylation at S727 happens in the nucleus and is necessary for maximal transcriptional activity^32^. Unlike IFNs, STAT1 phosphorylation in response to stress-inducers such as LPS and UV radiation results in direct phosphorylation of STAT1 only at S727^25^. This phosphorylation is dependent on p38. STAT1 phosphorylation at Y701 is not induced until later time points (2 h) and is not a direct result of the induced signaling cascade. In the case of LPS, phosphorylation at Y701 is dependent on IFN-$\upbeta$ induced on a feedback mechanism^33^.

Despite the similarity of LPS and gp96 induced phosphorylation of STAT1, there are notable and significant differences. Within BMDCs, LPS induces STAT1 S727 phosphorylation at 20 min and Y701 at 2 h. However, gp96 only induces phosphorylation at S727 at 2 h and Y701 is not observed at any time point tested. This indicates the signals emanating from CD91 and TLR4 are different prior to convergence at STAT1*.* One of these upstream differences is in the p38-dependence of the STAT1 phosphorylation at Y701. In both PECs and BMDCs, phosphorylation of STAT1 at S727 induced by gp96 is dependent on p38. In contrast, although phosphorylation of S727 by LPS in PECs is p38 dependent, this appears to be independent of p38 in BMDC at the 2 h time point. This could likely be due to the production of IFN-$\upbeta$ which is capable of phosphorylating STAT1 independent of p38^33^. There have been no reports of IFN-$\upbeta$ production following induction of cells by gp96 and thus, it is unclear if IFN-$\upbeta$ is a secondary mediator of STAT1 phosphorylation at the 2 h time points.

The responses of PECs and BMDCs have been largely similar in terms of CD91-dependent cross-presentation of gp96-chaperoned peptides, and provision of co-stimulation for T cell responses. Thus, gp96 primes T cell responses for anti-tumor immunity when mice are immunized either intraperitoneally (targeting PECs) or intradermally (targeting representative cDCs)^12^. Our data here and recent observations on the contributions of NK cell ‘helper’ activity to T cell responses^15,34^, present the importance of the APC in activating these effector cells. NK cells are activated by both cell surface and soluble ligands (IL-12, IL-15, and IL-18), depending on the immune microenvironment^35^. Soluble ligands activate both helper (IFN-$\gamma$) and lytic (granzyme) function in NK cells. In response to gp96, we have observed only NK cell helper activity^15^ and attribute this to at least CXCL10 released by PECs. Other soluble factors such as CXCL9, CXCL11, CCL2, CCL3, CCL4, and CCL5 have been shown to be able to activate NK cells, but less is known about the function of these activated NK cells^17,18^.

We have examined signaling cascades initiated by one immunogenic HSP, gp96, in the context of two representative APCs. The landscape becomes intricately more complicated by incorporating additional HSPs known to elicit immune responses following release from cells, into the picture. Calreticulin for example is known to stimulate IFN-$\upbeta$ in vivo^36^. This cytokine has a clear role in regulating STAT1 signaling. While we do observe IFN-$\gamma$ production by NK cells in PEC co-cultures activated with calreticulin (as well as HSP90), this HSP could trigger different signaling cascades from gp96 and potentially elicits NK cell activation through a different subset of cytokines and chemokines. In addition, at least 7 distinct CD91^+^ APCs are identifiable in the dermal/epidermal tissue alone. The holistic picture with multiple HSPs and multiple APCs will ultimately provide the immune signature that emanates from aberrant cells in pathologic tissues.

In response to gp96, APCs produce increased levels of both traditional cytokine activators such as IL-18, (but not IL-15 or IL-12) as well as the chemokines CXCL10 and CCL5. Although we demonstrate that NK cell activation is dependent upon CXCR3 ligands, it is not sufficient. Nor is the lack of CXCL10 expression the only deficiency in BMDCs preventing NK cell IFN-g production, as addition of exogenous CXCL10 in gp96-activated BMDC-NK cell co-cultures does not result in IFN-γ production (Supplemental Fig. 4). In addition, we do not exclude the possibility that other soluble or cell surface ligands are also involved, and that the pathway we’ve identified is one branch of a complex web. This is well illustrated when we use STAT1 KO PECs to activate NK cells. These PECs produce reduced levels of both CXCL10 and CCL5 which induce and inhibit IFN-$\gamma$ secretion, respectively. In addition, STAT1 is a prolific transcription factor and STAT1 KO PECs almost assuredly have differences in the production of other soluble and surface proteins, any of which could have a positive or negative effect on NK cell IFN-γ secretion. It is the net change from all of these differences that leads to increased IFN-$\gamma$ production by NK cells. CXCL10 is a known STAT1 induced gene and is required for NK cell activation. Our studies show that STAT1 phosphorylation is partially inhibited by increased levels of the phosphatase SHP2 in BMDCs. However, since abrogation of SHP2 activity does not fully restore NK cell activation properties to BMDCs in response to gp96, it implies SHP2 alone is not responsible for the differences observed between PECs and BMDCs.

Clinically, autologous tumor-derived gp96 has been administered subcutaneously for the therapeutic development of anti-tumor immunity in patients with melanoma, renal cell carcinoma and glioblastoma^37–39^. This route will target dermal DCs and macrophages and potentially those in the draining lymph nodes^9^. There has been no clinical study to date examining the targeted APCs or the contribution of NK cells to the overall anti-tumor immunity following gp96 administration subcutaneously or comparatively with other routes. Our study here places an emphasis on this mechanism and suggests that a rationale immunization regimen to maximize both NK cell and T cell effectors arms should be undertaken. This could be achieved by targeting APCs with specific immunization routes.

## Supplementary Information

Supplementary Information 1.

Supplementary Information 2.
