# Supplementary material for: Tunable heat shock protein-mediated NK cell responses are orchestrated by STAT1 in Antigen Presenting Cells
Source: Sci Rep. 2021 Aug 9;11:16106. doi: 10.1038/s41598-021-95578-3 (PMC8352880; doi:10.1038/s41598-021-95578-3)
Supplement: Supplementary file 1 — Supplementary Information 1. [file 41598_2021_95578_MOESM1_ESM.pdf]

Tunable Heat Shock Protein-Mediated NK cell responses are orchestrated by STAT1 in Antigen Presenting Cells

Abigail L. Sedlacek, Lauren B. Kinner-Bibeau, Yifei Wang, Alicia P. Mizes, Robert J. Binder. PhD

Supplemental Figures

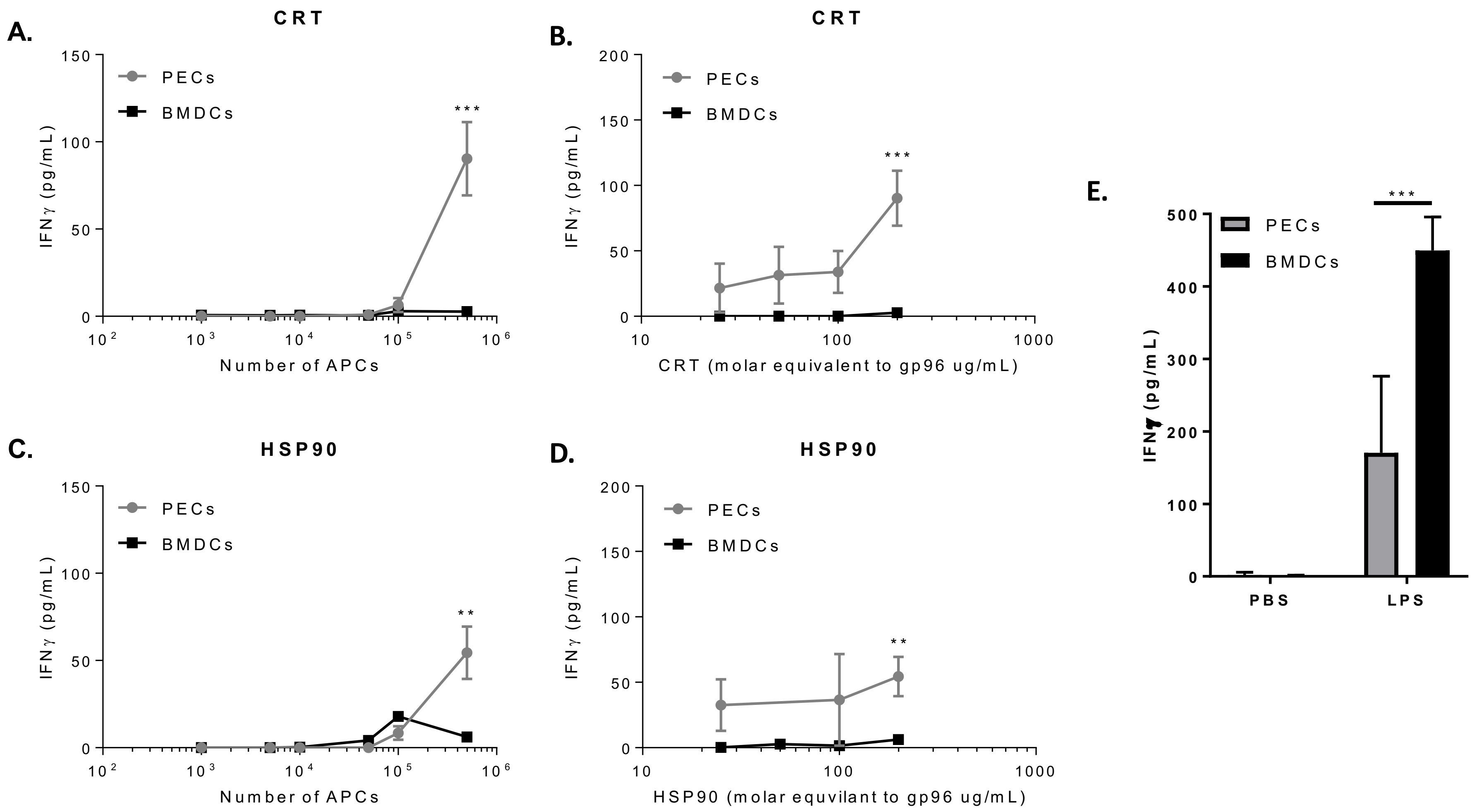

Sedlacek et al., Supplementary Figure 1.

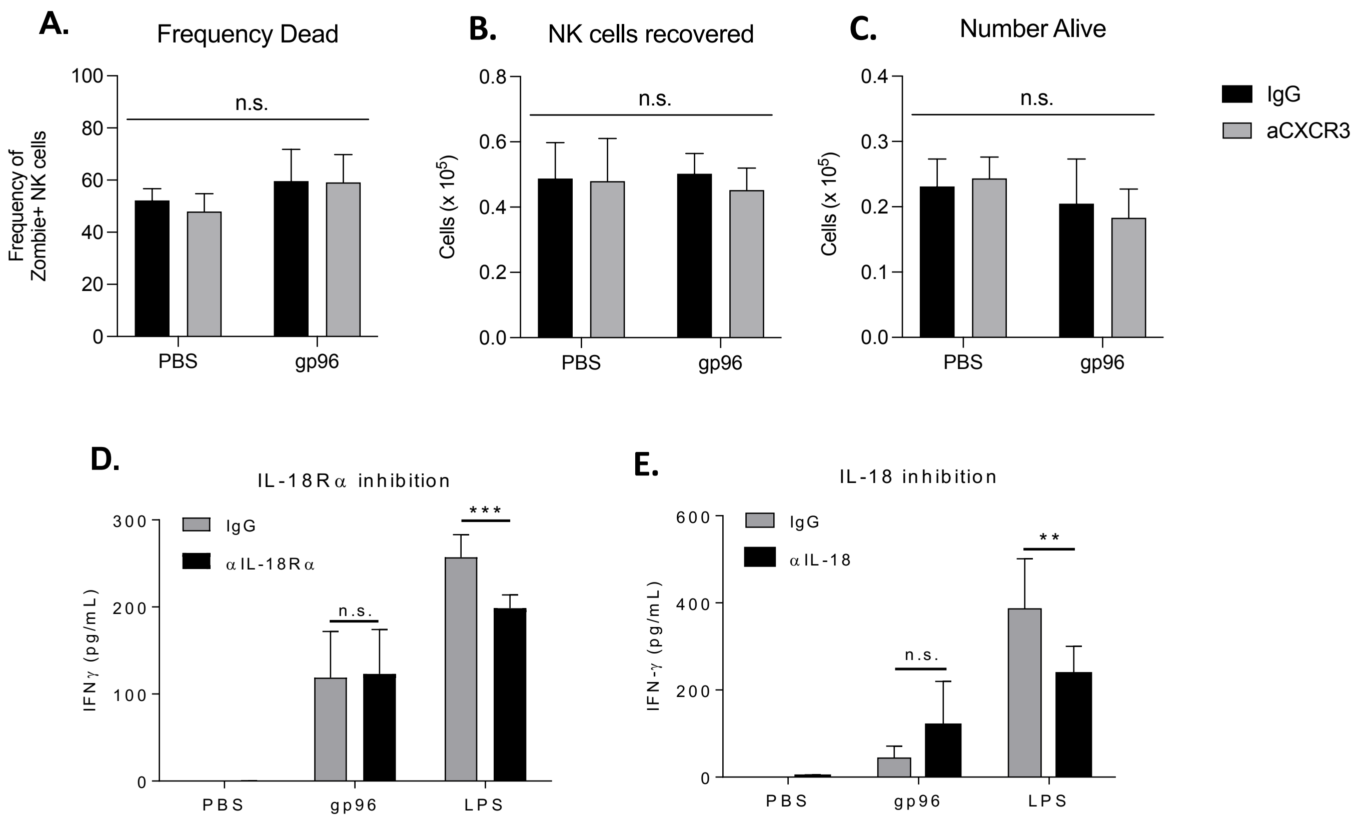

Sedlacek et al., Supplementary Figure 2.

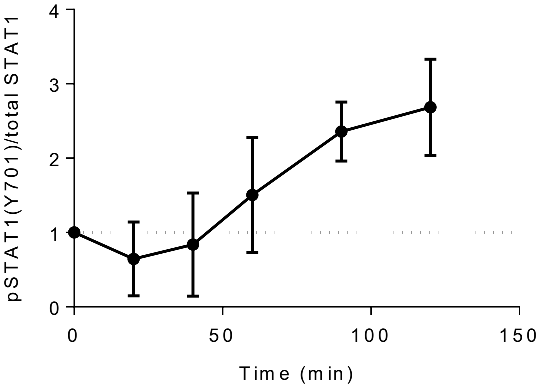

Sedlacek et al., Supplementary Figure 3.

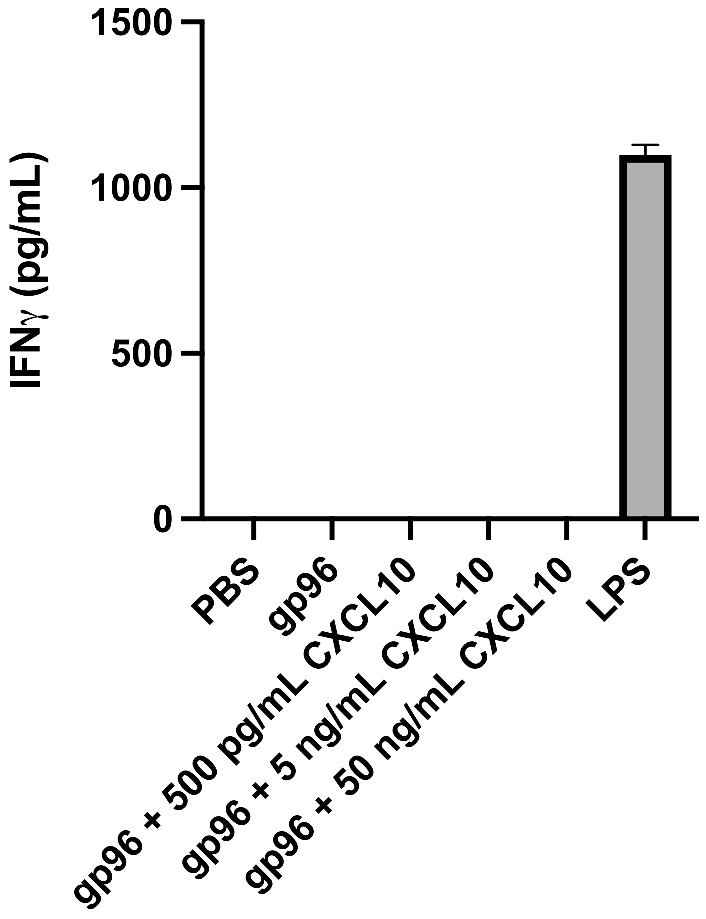

Sedlacek et al., Supplementary Figure 4.

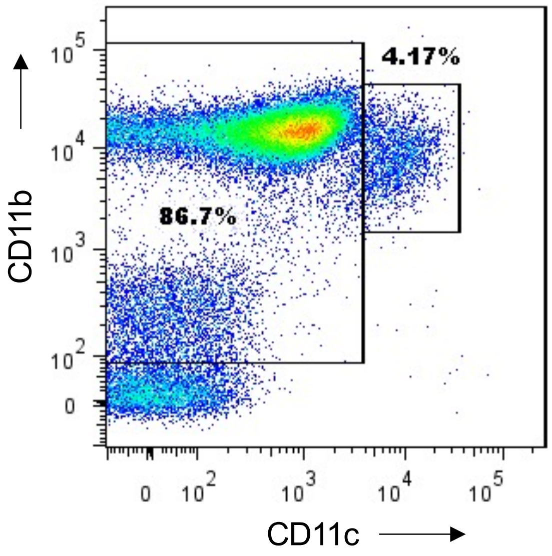

Sedlacek et al., Supplementary Figure 5.

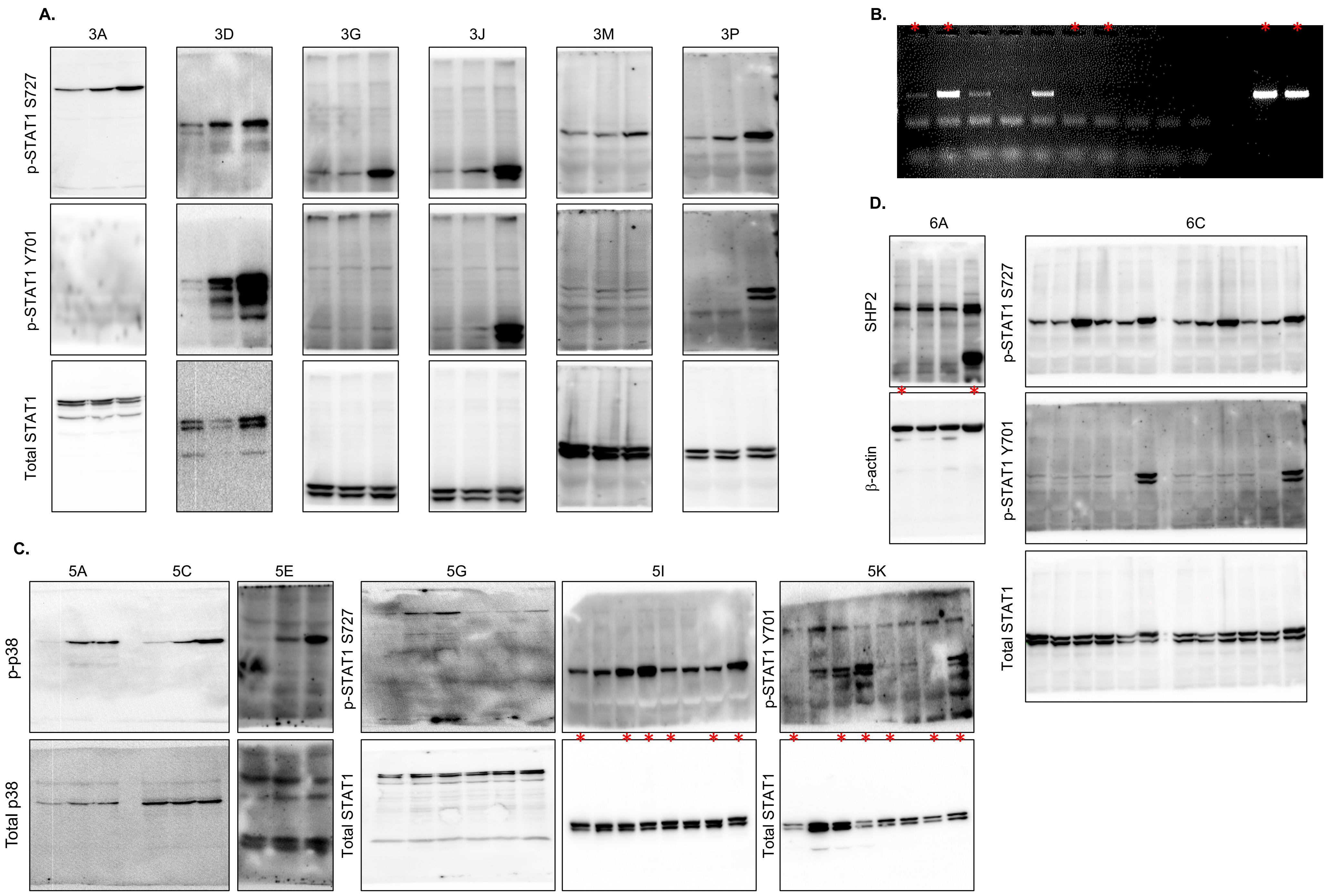

Sedlacek et al., Supplementary Figure 6.
