# Supplementary material for: Tunable heat shock protein-mediated NK cell responses are orchestrated by STAT1 in Antigen Presenting Cells
Source: Sci Rep. 2021 Aug 9;11:16106. doi: 10.1038/s41598-021-95578-3 (PMC8352880; doi:10.1038/s41598-021-95578-3)
Supplement: Supplementary file 2 — Supplementary Information 2. [file 41598_2021_95578_MOESM2_ESM.docx]

**Supplemental Figure 1. Calreticulin and HSP90 induce IFN-γ production preferentially in PEC-NK cell co-cultures.** Equivalent numbers of PECs or BMDCs were co-cultured with 5x10^4^ NK cells and the indicated dose of **(A and B)** calreticulin (CRT), **(C and D)** HSP90, or **(E)** LPS for 24 hours. Supernatants were assayed for IFN-γ secretion by ELISA. **(A and C)** Numbers of antigen presenting cells were titrated using a constant concentration of **(A)** CRT or **(C)** HSP90. The dose of CRT and HSP90 used is the molar equivalent of 200ug/mL gp96. **(B and D)** Concentration of **(B)** CRT or **(D)** HSP90 was titrated using a constant (5x10^5^) number of APCs. **(E)** Co-cultures with 5x10^5^ APCs were activated with 1μg LPS. Data are represented as mean ± s.d, **p<0.01, ***p<0.001 (Student’s t-test).

**Supplemental Figure 2. Anti-CXCR3 does not induce NK cell death and IL-18 is not required for indirect gp96 mediated NK cell activation.** One hundred and fifty thousand PECs (5x10^4^ adherent cells) were plated with 5x10^4^ NK cells in the presence of 200 μg/mL gp96 and **(A-C)** α-CXCR3 antibody or **(D)** α-IL-18Rα antibody or **(E)** α-IL-18 antibody **(A-C)** NK cells were harvested after 72 hours, stained with Zombie UV, and analyzed by flow. **(A)** Frequency of Zombie positive (dead) cells among the NK cell population. **(B)** Cell count of NK cells recovered from the co-culture assay. **(C)** Using the number of NK cells recovered and the frequency of Zombie negative (live) cells, the number of live cells recovered was calculated **(D-E)** Supernatant was harvested after 72 hours and assayed by IFN-γ ELISA. Data in both panels are pooled from 3 independent experiments. Data are represented as mean ± s.d. *ns* not significant, *p<0.05, **p<0.01, ***p<0.001 (Two-way ANOVA with Sidak’s multiple comparison test)

**Supplemental Figure 3. Time course of pSTAT1 Y701 phosphorylation in PECs treated with 200 μg/mL gp96.** PECs were activated with 200 μg/mL gp96 over several time points. Cells lysates were obtained, analyzed by SDS-PAGE and immunoblotted for total STAT1 and phospho-STAT1 at residue Y701. STAT1 phosphorylation at Y701 is plotted over time each data point is pooled from at least 3 independent experiments. Data are represented as mean ± s.d.

**Supplemental Figure 4. Addition of CXCL10 to BMDC-NK cell co-cultures does not induce IFN-γ production.** BMDCs and NK cells were co-cultured with 200ug/mL gp96 and the indicated amount of CXCL10 for 72 hours. Supernatant was harvested and assayed by IFN-γ ELISA. LPS was used as a positive control.

**Supplemental Figure 5. Representative flow cytometry analysis of adherent PECs.** Total PECs were plated overnight and non-adherent cells were washed away. Adherent cells were trypsinized and stained for flow cytometry with α-CD11b and α-CD11c.

**Supplemental Figure 6. Uncropped Immunoblot Images.** A. Corresponds to the immunoblots in Figure 3 as indicated. B. Corresponds to the DNA gels in Figure 4. Corresponds to the immunoblots in Figure 5. C. Corresponds to the immunoblots in Figure 6. * indicates relevant lanes in blots when extraneous lanes were excluded.
